# The Association of Serum Immunoglobulins with Risk of Cardiovascular Disease and Mortality: the Rotterdam Study

**DOI:** 10.1007/s10875-023-01433-7

**Published:** 2023-02-01

**Authors:** Samer R. Khan, Virgil A. S. H. Dalm, M. Kamran Ikram, Robin P. Peeters, P. Martin van Hagen, Maryam Kavousi, Layal Chaker

**Affiliations:** 1 Department of Internal Medicine, Division of Allergy & Clinical Immunology, Erasmus University Medical Center Rotterdam, Rotterdam, the Netherlands; 2 Department of Epidemiology, Erasmus University Medical Center Rotterdam, Doctor Molewaterplein 50, 3015 GE Rotterdam, the Netherlands; 3 Department of Immunology, Erasmus University Medical Center Rotterdam, Rotterdam, the Netherlands; 4 Department of Neurology, Erasmus University Medical Center Rotterdam, Rotterdam, the Netherlands; 5 Department of Internal Medicine, Division of Endocrinology, Erasmus University Medical Center Rotterdam, Rotterdam, the Netherlands

**Keywords:** Atherosclerosis, myocardial infarction, myocardial revascularization, stroke, mortality, immunoglobulins

## Abstract

**Purpose:**

Inflammation is implicated in cardiovascular disease (CVD), but the association of total serum immunoglobulin (Ig) A, G, and M with CVD across the whole spectrum of atherosclerosis in community-dwelling elderly is unknown.

**Methods:**

This study was embedded in the Rotterdam Study, an ongoing population-based cohort study. We performed Cox regression for the associations of Igs with incident atherosclerotic CVD (ACVD; composite of myocardial infarction, revascularization, and stroke), cardiovascular mortality, and all-cause mortality, and multinomial logistic regression for the association between Igs and coronary artery calcification (CAC) scores. We adjusted for age, sex, lifestyle, and cardiovascular risk factors and presented results per standard deviation increase.

**Results:**

We included 8767 participants (median age 62.2 years, 57% women). Higher IgG was associated with an increased ACVD risk (hazard ratio [HR]: 1.08; 95% confidence interval [95% CI]: 1.01–1.15). Higher IgA and IgG were associated with an increased cardiovascular mortality risk, mainly within Ig reference ranges, and with an increased all-cause mortality risk, although less marked. Higher IgA was associated with severe atherosclerosis, i.e., CAC score > 400 (odds ratio: 1.29; 95% CI: 1.03–1.62), while for higher IgG a trend was seen with severe atherosclerosis.

**Conclusion:**

In middle-aged and older individuals from the general population, higher serum IgA and IgG, but not IgM, are associated with CVD, cardiovascular mortality, and severe atherosclerosis, particularly within Ig reference ranges and independent of serum C-reactive protein. Future studies are needed to elucidate potential causality of the reported associations.

**Supplementary Information:**

The online version contains supplementary material available at 10.1007/s10875-023-01433-7.

## Introduction

Cardiovascular disease (CVD) is the main cause of disability and mortality worldwide with atherosclerosis as the most common underlying pathology [1, 2]. The etiology of atherosclerosis is complex but involves several metabolic and inflammatory factors [2, 3]. Coronary artery calcification (CAC), considered an early manifestation of atherosclerosis, is positively correlated with the extent of atherosclerosis and related to inflammation as well [4]. Recently, several components of the immune system have been targeted in an attempt to reduce inflammation leading to atherosclerosis with anti-interleukin-1 (IL-1) and anti-interleukin-6 (IL-6) targeted therapy showing promising results [5, 6].

In contrast, the contribution of immunoglobulins (Igs) to atherosclerosis has been relatively understudied [2, 3]. In mouse models, a decreased number of B2-cells due to B-cell activating factor receptor deficiency, resulting in diminished levels of IgG1, IgG2a, and IgM, was associated with less atherosclerosis [7]. However, reviews of studies mainly performed in rodents reported inconclusive results on the effects of serum Igs depending on the type of Ig. IgA and IgG may generally have a stimulating effect on atherosclerosis, e.g., via reaction with atherothrombotic or oxidation-specific epitopes, whereas IgM may be predominantly protective through neutralization of atherosclerotic antigens [8, 9].

Reviews of studies in humans reported various IgA and IgG auto-antibodies that were mostly associated with an increased risk of CVD, whereas results were inconclusive for the association between IgM auto-antibodies and CVD risk [10, 11]. Pathogen-specific antibodies have been related to CVD as well and over the years multiple trials were performed to assess the efficacy of antibiotic treatment in the prevention of CVD and cardiovascular mortality, but utility could not be demonstrated [12, 13]. However, studies on the association between total serum levels of Igs and CVD are sparse and show inconclusive results, while total serum Ig measurements are readily available in clinical practice and may provide a broader overview of the immune system status in the context of atherosclerosis. Two studies showed a relation between higher Ig levels and CVD [14, 15], while two other studies reported a relation between higher Ig levels and a lower risk of CVD or less severe cardiovascular outcomes [16, 17]. However, these studies were either cross-sectional or did not include time-to-event analyses, did not adjust for potential confounders including cardiovascular risk factors, or categorized serum Ig levels resulting in loss of information. One longitudinal population-based study failed to show an association between Igs and CVD, which could be due to being underpowered with a total of 47 nonfatal and 10 fatal ischemic events. Furthermore, the study was set in a different cardiovascular risk management era, with blood drawn in 1984 and four years of follow-up [18]. Due to the design of these previous studies, reverse causation cannot be ruled out. Furthermore, most studies were patient-based with mainly or exclusively male participants, thus limiting generalizability of results, and none of the studies investigated the association of Igs with subclinical atherosclerosis.

We performed the current study to address abovementioned limitations of previous studies in order to shed more light on the relation between Igs and the whole spectrum of atherosclerosis in individuals from the general population. To this end, we studied the association of serum Igs with atherosclerotic CVD (ACVD; composite of myocardial infarction, revascularization, and stroke), cardiovascular (both atherosclerotic and non-atherosclerotic) mortality, and CAC scores in a population-based cohort of middle-aged and older individuals with over 17 years of follow-up. We adjusted for serum C-reactive protein (CRP) to establish whether the association of serum Igs with cardiovascular outcomes is independent of currently known inflammatory pathways including CRP and ILs. Furthermore, we assessed the association between serum Igs and all-cause mortality to investigate potential differences that may proxy the relation of Igs with cardiovascular compared to systemic inflammation.

## Methods

### Study Design and Participants

This study was embedded in the Rotterdam Study (RS), an ongoing prospective population-based cohort study including middle-aged and older inhabitants of Ommoord, Rotterdam, the Netherlands. The RS started in 1990 and included interested inhabitants of the study area that were aged 55 years or over. The RS was extended with additional independent cohorts in 2000 and 2006 and for these cohorts, inhabitants aged 55 years or over (RS cohort II) respectively 45 years or over (RS cohort III) that were not previously invited were invited to participate. By the end of 2008, 14,926 participants aged 45 years or over had been enrolled (response rate 72%). In 2016, enrollment for a fourth cohort started aimed at inhabitants of the study area aged 40 years or over. After study entry, medical records of general practitioners (GPs) are continuously linked to the RS database and outcomes of interest are validated according to standardized guidelines by medical experts in the field. In addition, each participant is re-examined every three to six years at the research center in Ommoord and through home interviews. The RS complies with the declaration of Helsinki and has been approved by the medical ethics committee of the Erasmus University Medical Center (registration number MEC 02.1015) and by the Dutch Ministry of Health, Welfare and Sport (Population Screening Act WBO, license number 1071272–159,521-PG). The RS has been entered into the Netherlands National Trial Register and into the WHO International Clinical Trials Registry Platform under shared catalogue number NTR6831. More detailed information can be found elsewhere [19].

For this study, we included participants of three independent RS cohorts (RS I-3, II-1, and III-1) with written informed consent for follow-up, available measurements of serum IgA, IgG, and/or IgM at study baseline, and information on cardiovascular outcomes of interest (*n* = 8767).

### Assessment of Serum Igs

Blood was drawn at the research center through 1997–1999 (RS cohort I-3), 2000–2001 (RS cohort II-1), and 2006–2008 (RS cohort III-1) and the moment of blood drawing was considered the study baseline. Serum samples were subsequently stored at − 80 °C. Serum IgA, IgG, and IgM measurements took place between 2016 and 2018 through an immunoturbidimetric assay (Tina-quant® IgA/IgG/IgM Gen. 2, Roche Diagnostics GmbH, Mannheim, Germany). Recommended reference ranges according to the manufacturer’s protocol were 0.7–4.0 g/L for IgA, 7.0–16.0 g/L for IgG, and 0.4–2.3 g/L for IgM. As previously described, reference ranges based on 2.5^th^ and 97.5^th^ percentiles for the RS population were 0.86–4.76 g/L for IgA, 6.20–15.10 g/L for IgG, and 0.28–2.64 g/L for IgM [20].

### Assessment of ACVD, Cardiovascular and All-Cause Mortality, and CAC

ACVD was defined as myocardial infarction, revascularization (percutaneous coronary intervention or coronary artery bypass graft), or stroke in accordance with previous research and specific guidelines [21]. Information on prevalent (at baseline) ACVD was retrieved through home interviews, linkage with the Nationwide Medical Registry (a national registry of all hospital discharge diagnoses of all Dutch inhabitants), and medical records of GPs. Information on incident (during follow-up) ACVD was retrieved through continuous automated linkage with medical records of GPs containing ICPC codes as diagnosed by GPs or medical specialists and was subsequently validated as described previously [22–24]. Follow-up of myocardial infarction, revascularization, and the composite endpoint of ACVD was complete until January 1, 2015. Follow-up of stroke (comprising all stroke cases, i.e., ischemic, hemorrhagic, and unspecified) was complete until January 1, 2016.

Information on mortality was retrieved from medical records of GPs, hospitals, and nursing homes. Two independent research physicians classified mortality according to ICPC and ICD-10 codes. Subsequently, all coded events were reviewed by a senior physician in the field to confirm the diagnosis. Date of death was retrieved from the medical records or municipality records. Cardiovascular mortality was composed of atherosclerotic and non-atherosclerotic mortality. Atherosclerotic mortality was defined as mortality due to coronary heart disease, cerebrovascular disease, or other atherosclerotic disease (ICPC codes K75-K77, K90-K92; ICD-10 codes I21-I25, I50, I60-I70, I71.3, I71.4, I73-I74, I77-I79, Y60.5) and non-atherosclerotic mortality was defined as mortality due to other cardiovascular disease (ICPC codes K78-K87, K93-K94, K99; ICD-10 codes I05-I13, I15, I26-I28, I30-I49, I51-I52, I71[excluding I71.3 and I71.4]-I72, I80-I83, I97-I99, T82) in accordance with previous literature [22]. Follow-up of cardiovascular mortality was complete until January 1^st^ 2015. All-cause mortality comprised any (both cardiac and non-cardiac) mortality and its follow-up was complete until May 24, 2018.

CAC scores were available in a random subset of RS cohorts I-3 and II-1 (*n* = 1622) and were measured by electronbeam computed tomography (EBT; C-150 Imatron Scanner, GE Healthcase, South San Francisco, CA). Participants had to lie still and hold their breath during the assessment. Thirty-eight images were obtained from the aorta root to the heart with 100-ms scan time and 3-mm slice thickness. CAC was quantified with AccuImage software (AccuImage Diagnostics Corp), displaying all pixels with a density of > 130 Hounsfield units (HU). Calcification was defined as ≥ 2 adjacent pixels of > 130 HU. CAC scores were calculated by multiplication of the area of individual calcifications in mm^2^ with a factor based on the peak density of the calcification. All individual CAC scores were combined to obtain a total CAC score for the entire coronary epicardial system. Assessment of CAC was performed by two independent experienced physicians [25].

### Assessment of Baseline Covariates

Weight, height, and blood pressure were measured at the research center in Ommoord. Body mass index (BMI) was defined as weight divided by height squared (kg/m^2^). Blood pressure was measured twice at the right brachial artery with the participant in sitting position. The average of these two consecutive measurements was taken. Hypertension was defined as a blood pressure exceeding 140/90 mmHg or as the use of blood pressure lowering medication with the indication of hypertension. Information on baseline type 2 diabetes (DM) was collected from GPs and pharmacies and assessed through blood samples collected at the research center. DM was defined as a fasting blood glucose concentration of ≥ 7.0 mmol/L, a non-fasting blood glucose concentration of ≥ 11.1 mmol/L (when fasting samples were unavailable), or as the use of blood glucose-lowering drugs or dietary treatment for diabetes. Smoking status, alcohol consumption, and highest attained education (as proxy for socioeconomic status) were assessed through questionnaires during home interviews. Smoking status was defined as never, former, or current smoker. Alcohol consumption was reported in gram/day and categorized into none, mild (0–10 g/day), moderate (10–20 g/day), or heavy (> 20 g/day). Physical activity was assessed using validated questionnaires and expressed in standardized metabolic equivalent of task (MET) hours/week [26, 27]. Serum triglycerides and total cholesterol (mmol/L) were measured using an automated enzymatic procedure. Serum CRP (mg/L) was measured with an immunoturbidimetric assay. Baseline use of medication known to potentially alter serum Ig levels (systemic corticosteroids, antiepileptic drugs, angiotensin converting enzyme inhibitors, cytostatics, immunomodulators, and/or immunosuppressants) was established during home interviews and coded based on the Anatomical Therapeutic Chemical Classification System. None of the participants used intravenous or subcutaneous Igs.

### Statistical Analyses

Serum Igs were standardized for all analyses to facilitate comparison of results. In the longitudinal analyses, participants were followed until the first event of interest, death, or end of follow-up, whichever came first. All analyses included three models, adjusting for potential confounders based on biological plausibility and previous comparable research [21]. In the first model, we adjusted for age and sex. In the second model, we additionally adjusted for smoking status, alcohol consumption, physical activity, and highest attained education. The third model included confounders that could also act as mediators and comprised BMI, DM, hypertension, serum triglycerides, CRP, and total serum cholesterol additional to the first two models. The longitudinal analyses also included RS cohort in all models to take temporal trends into account.

The association between serum Igs and incident ACVD (both composite and the individual endpoints of myocardial infarction, revascularization, and stroke) was assessed through Cox proportional hazards regression analyses after exclusion of participants with prevalent ACVD. The proportional hazards assumption was checked through the Schoenfeld test and plot and was met for all analyses. A sensitivity analysis was performed by excluding participants with serum Ig levels outside our calculated reference ranges (0.86–4.76 g/L for IgA, 6.20–15.10 g/L for IgG, and 0.28–2.64 g/L for IgM) and users of medication known to potentially alter serum Ig levels in order to limit the influence of transient outliers in serum Ig levels. With respect to stroke, we also examined the association of Igs with ischemic stroke.

The association of serum Igs with cardiovascular mortality (atherosclerotic, non-atherosclerotic, and combined) and all-cause mortality was assessed by Cox proportional hazards regression analyses. Proportional hazards were checked and confirmed for all analyses. For cardiovascular mortality, we stratified by prevalent ACVD status, age (cut-off 65 years), and sex, and performed a sensitivity analysis after exclusion of participants with serum Ig levels outside the reference range and users of medication known to potentially alter serum Ig levels. For comparison, we applied both the assay recommended and our own calculated reference ranges in this sensitivity analysis. We furthermore displayed the risk of cardiovascular mortality for participants with the highest compared to the lowest Ig reference value (4.76 vs 0.86 g/L for IgA, 15.10 vs 6.20 g/L for IgG, and 2.64 vs 0.28 g/L for IgM) while keeping all other covariates constant by testing contrasts of the Cox proportional hazards regression analyses.

For the association between serum Igs and CAC score, we categorized CAC scores into no (score = 0), mild (score 0–100), moderate (score 100–400), or severe calcification (score > 400) based on the most commonly used classification system [28]. The association between serum Igs and CAC score categories was assessed through multinomial logistic regression analyses, while taking no calcification as the reference category. There was no multicollinearity and the linearity of log odds assumption was met. We performed a sensitivity analysis by excluding participants with serum Ig levels outside abovementioned calculated reference ranges and users of medication known to potentially alter serum Ig levels.

Missing values in covariates were imputed with multivariate imputation by chained Eqs. (4 imputations, 10 iterations). Missingness was < 2% for all covariates, except for physical activity and alcohol consumption (14.1% and 20.4% respectively). Convergence was reached and the distribution of covariates before and after imputation was similar. All analyses were performed with R Statistical Software version 4.0.2. Provided that effect estimates of the second and third models were comparable, we described the fully adjusted models in the results, unless stated otherwise.

## Results

We included 8,767 participants with a median age of 62.2 years and of whom 57% were women. Median values of IgA, IgG, and IgM were 2.10, 9.70, and 0.85 g/L respectively. Standard deviations (SDs) were 1.06 for IgA, 2.39 for IgG, and 0.98 for IgM. At baseline, 822 (9.4%) participants had prevalent ACVD. Baseline characteristics are displayed in Table 1. In participants without prevalent ACVD, 1020 incident ACVD events occurred during a median follow-up of 8.3 years (interquartile range [IQR]: 6.8–14.0), with an incidence rate of 13.4 per 1000 person-years. A total of 655 events occurred of cardiovascular mortality (median follow-up 8.6 years; IQR: 7.0–14.2) and 2,992 events of all-cause mortality (median follow-up 10.0 years; IQR: 7.7–15.4). Completeness of follow-up for cardiovascular mortality was 99.2% [29].

**Table 1:** Baseline characteristics of 8767 participants with Ig measurements and informed consent for follow-up

Sex, women, *n* (%)	4994 (57.0)
Age, years, median (IQR)	62.2 (57.5 − 70.7)
Serum IgA, g/L, median (IQR)	2.10 (1.57 − 2.78)
Serum IgG, g/L, median (IQR)	9.70 (8.30 − 11.20)
Serum IgM, g/L, median (IQR)	0.85 (0.59 − 1.23)
BMI, kg/m^2^, median (IQR)	26.8 (24.5 − 29.6)
Hypertension, *n* (%)	5346 (70.0)
Diabetes mellitus, *n* (%)	1040 (11.9)
Stroke, *n* (%)	302 (3.4)
Revascularization, *n* (%)	337 (3.8)
Myocardial infarction, *n* (%)	404 (4.6)
Coronary artery calcification score, median (IQR)^a^	106.5 (9.28 − 484.8)
Smoking status, *n* (%) - Never - Former - Current	- 2894 (33.0) - 4161 (47.5) - 1712 (19.5)
Alcohol consumption, *n* (%) - None - Mild (0 − 10 g/day) - Moderate (10 − 20 g/day) - Heavy (> 20 g/day)	- 1550 (17.7) - 4725 (53.9) - 1427 (16.3) - 1065 (12.1)
Standardized physical activity, MET hours/week, median (IQR)	− 0.16 (− 0.67–0.52)
Highest attained education, n (%) - Primary education - Lower/intermediate general education OR lower vocational education - Intermediate vocational education OR higher general education - Higher vocational education OR university	- 1049 (12.0) - 3522 (40.2) - 2540 (29.0) - 1656 (18.9)
Serum cholesterol, mmol/L, mean (SD)	5.70 (1.03)
Serum triglycerides, mmol/L, median (IQR)	1.33 (1.00 − 1.82)
Serum CRP, mg/L, median (IQR)	1.50 (0.60 − 3.40)
Use of immunomodulating medication^b^, *n* (%)	1374 (15.7)

*BMI* body mass index, *CRP* C-reactive protein, *Ig* immunoglobulin, *IQR* interquartile range, *MET* metabolic equivalent of task, *SD* standard deviation

^a^Available in a random subset of the study population (*n* = 1622)

^b^Comprises systemic corticosteroids, antiepileptic drugs, angiotensin-converting enzyme inhibitors, cytostatics, immunomodulators, and/or immunosuppressants

### Association Between Serum Igs and Incident ACVD

IgA was not associated with the composite outcome of incident ACVD or with myocardial infarction, revascularization, or stroke separately (Table 2). Higher IgG was associated with a modestly increased risk of ACVD (hazard ratio [HR] per SD: 1.08; 95% confidence interval [95% CI]: 1.01–1.15) and this was driven by an increased risk of myocardial infarction and revascularization. IgM was not associated with incident ACVD (Table 2).

**Table 2:** Association between immunoglobulins per SD and risk of ACVD

		Hazard Ratio (95% Confidence Interval)		
	*N events/total*	*Model 1*	*Model 2*	*Model 3*
**All**				
IgA	1020/7852	1.01 (0.95 − 1.07)	1.03 (0.97 − 1.09)	1.02 (0.96 − 1.09)
IgG	1019/7842	1.04 (0.98 − 1.11)	1.07 (1.00 − 1.14)	1.08 (1.01 − 1.15)
IgM	1020/7849	1.00 (0.96 − 1.05)	1.00 (0.96 − 1.05)	1.01 (0.96 − 1.06)
**Myocardial Infarction**				
IgA	421/8342	0.94 (0.85 − 1.04)	0.96 (0.86 − 1.06)	0.95 (0.86 − 1.06)
IgG	421/8332	1.04 (0.95 − 1.15)	1.07 (0.97 − 1.18)	1.08 (0.97 − 1.19)
IgM	421/8338	1.02 (0.96 − 1.08)	1.02 (0.96 − 1.08)	1.02 (0.96 − 1.09)
**Revascularization**				
IgA	414/8332	0.97 (0.88 − 1.08)	0.99 (0.90 − 1.10)	0.98 (0.88 − 1.09)
IgG	414/8322	1.05 (0.95 − 1.17)	1.08 (0.98 − 1.20)	1.08 (0.98 − 1.20)
IgM	414/8328	1.01 (0.93 − 1.09)	1.01 (0.93 − 1.09)	1.02 (0.94 − 1.10)
**Stroke**				
IgA	601/8464	1.03 (0.96 − 1.12)	1.04 (0.97 − 1.13)	1.04 (0.96 − 1.12)
IgG	600/8454	0.98 (0.90 − 1.07)	1.01 (0.93 − 1.10)	1.01 (0.93 − 1.10)
IgM	601/8461	0.98 (0.91 − 1.06)	0.98 (0.91 − 1.05)	0.99 (0.92 − 1.06)

Model 1 is adjusted for age, sex, and Rotterdam Study cohort; model 2 is adjusted for model 1, smoking status, alcohol consumption, physical activity, and highest attained education; model 3 is adjusted for model 2, body mass index, diabetes mellitus, hypertension, serum cholesterol, serum C-reactive protein, and serum triglycerides. *ACVD* atherosclerotic cardiovascular disease, *IgA*, immunoglobulin A, *IgG* immunoglobulin G, *IgM* immunoglobulin M, *SD* standard deviation

Effect estimates did not materially change after exclusion of participants with Ig levels outside the reference range and users of potentially Ig-altering medication (Supplementary Table S1). Effect estimates for the association of serum Igs with ischemic stroke were comparable to those with all stroke (Supplementary Table S2).

### Association Between Serum Igs and Cardiovascular and All-Cause Mortality

Higher IgA was associated with an increased risk of all cardiovascular (HR: 1.13 per SD; 95% CI: 1.06–1.21) and atherosclerotic cardiovascular mortality (HR: 1.14 per SD; 95% CI: 1.05–1.23). The association between higher IgA and non-atherosclerotic cardiovascular mortality did not reach statistical significance (HR per SD: 1.11; 95% CI: 0.98–1.25) (Table 3). Higher IgG was associated with all cardiovascular (HR: 1.14 per SD; 95% CI: 1.07–1.22), atherosclerotic (HR: 1.12 per SD; 95% CI: 1.04–1.22), and non-atherosclerotic cardiovascular mortality (HR: 1.19 per SD; 95% CI: 1.05–1.34). IgM was not associated with cardiovascular mortality risk (Table 3).

**Table 3:** Association between immunoglobulins per SD and risk of cardiovascular mortality

	Full range				Reference range^a^			
		Hazard ratio (95% confidence interval)				Hazard ratio (95% confidence interval)		
	*N events/total*	*Model 1*	*Model 2*	*Model 3*	*N events/total*	*Model 1*	*Model 2*	*Model 3*
**All cardiovascular mortality**								
IgA	655/8766	1.13 (1.06 − 1.21)	1.14 (1.07 − 1.21)	1.13 (1.06 − 1.21)	426/7,037	1.15 (1.02 − 1.29)	1.17 (1.04 − 1.31)	1.18 (1.04 − 1.33)
IgG	655/8756	1.12 (1.05 − 1.20)	1.15 (1.07 − 1.22)	1.14 (1.07 − 1.22)	424/7,036	1.19 (1.06 − 1.35)	1.24 (1.10 − 1.40)	1.24 (1.09 − 1.39)
IgM	655/8762	1.01 (0.96 − 1.06)	1.01 (0.95 − 1.06)	1.01 (0.96 − 1.07)	426/7,073	1.06 (0.87 − 1.29)	1.08 (0.89 − 1.32)	1.08 (0.89 − 1.32)
**Atherosclerotic cardiovascular mortality**								
IgA	455/8766	1.14 (1.05 − 1.23)	1.14 (1.05 − 1.23)	1.14 (1.05 − 1.23)	301/7,037	1.12 (0.97 − 1.29)	1.13 (0.98 − 1.31)	1.15 (0.99 − 1.32)
IgG	455/8,756	1.10 (1.01 − 1.19)	1.13 (1.04 − 1.22)	1.12 (1.04 − 1.22)	304/7,036	1.19 (1.03 − 1.37)	1.23 (1.07 − 1.42)	1.23 (1.06 − 1.42)
IgM	455/8762	1.01 (0.95 − 1.08)	1.00 (0.94 − 1.07)	1.01 (0.95 − 1.08)	305/7,073	0.97 (0.77–1.24)	1.00 (0.78–1.27)	1.00 (0.78–1.27)
**Non-atherosclerotic cardiovascular mortality**								
IgA	200/8766	1.12 (0.99–1.26)	1.12 (0.99–1.27)	1.11 (0.98–1.25)	125/7,037	1.22 (0.99–1.52)	1.25 (1.01–1.55)	1.26 (1.01–1.56)
IgG	200/8756	1.19 (1.05–1.34)	1.20 (1.06–1.34)	1.19 (1.05–1.34)	120/7,036	1.21 (0.97–1.51)	1.24 (1.00–1.55)	1.25 (1.00–1.56)
IgM	200/8762	1.00 (0.91–1.11)	1.00 (0.91–1.11)	1.01 (0.91–1.12)	121/7,073	1.29 (0.90–1.83)	1.30 (0.91–1.84)	1.31 (0.92–1.86)

^a^Comprises reference range values (0.86–4.76 g/L for IgA, 6.20–15.10 g/L for IgG, and 0.28–2.64 g/L for IgM) and exclusion of medication use that can influence serum immunoglobulins (systemic corticosteroids, antiepileptic drugs, angiotensin converting enzyme inhibitors, cytostatics, immunomodulators, and/or immunosuppressants)

Model 1 is adjusted for age, sex, and Rotterdam Study cohort; model 2 is adjusted for model 1, smoking status, alcohol consumption, physical activity, and highest attained education; model 3 is adjusted for model 2, body mass index, diabetes mellitus, hypertension, serum cholesterol, serum C-reactive protein, and serum triglycerides. *IgA* immunoglobulin A, *IgG* immunoglobulin G, *IgM* immunoglobulin M; *SD* standard deviation

Effect sizes for higher IgA and IgG increased after exclusion of participants with Ig levels outside the reference range and users of potentially Ig-altering medication (Table 3). Results after exclusion of participants with Ig levels outside assay recommended reference ranges were comparable to results after exclusion of participants with Ig levels outside our calculated reference ranges (Table 3, Supplementary Table S3).

**Fig. 1:**
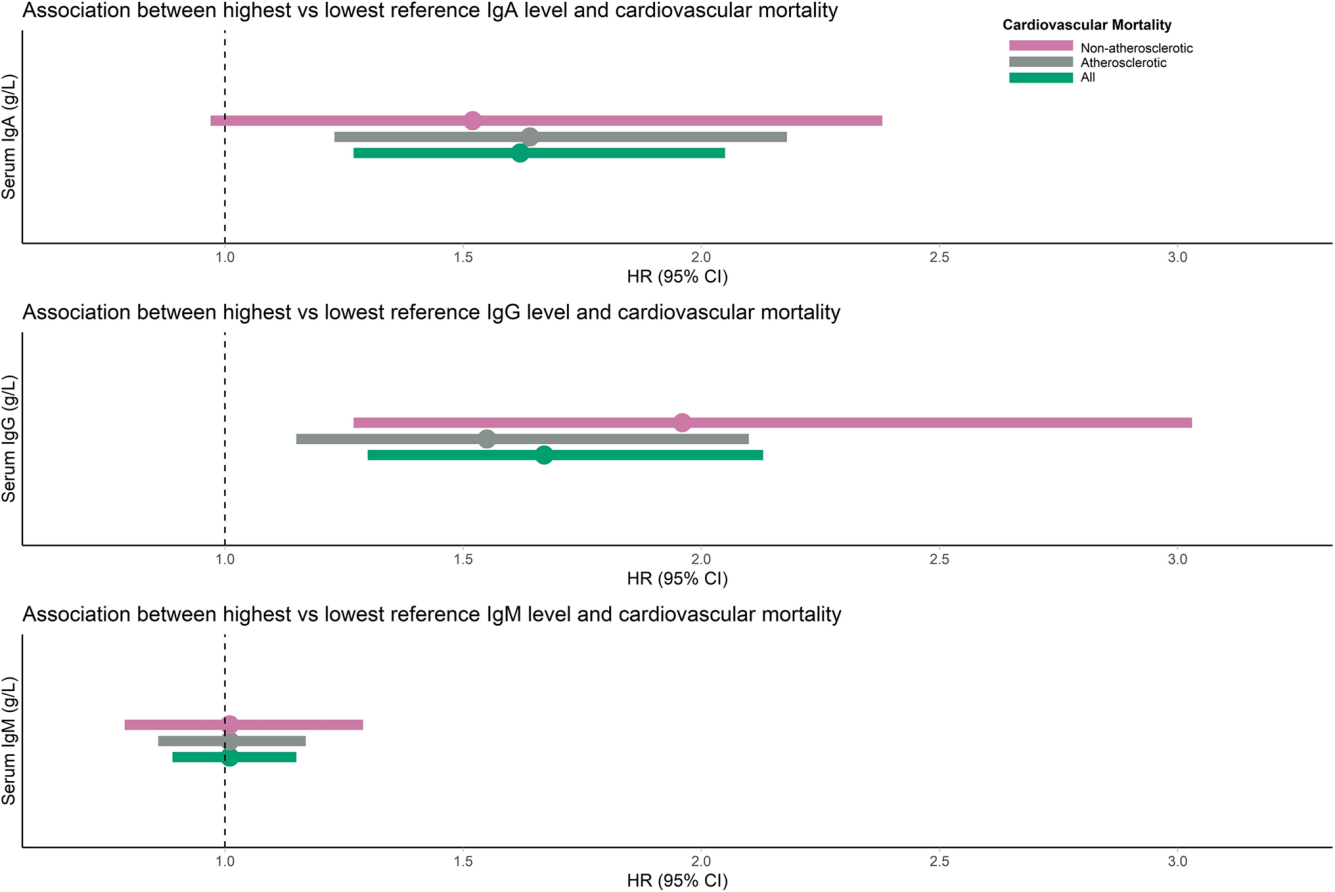
Association between highest compared to lowest reference immunoglobulin levels and risk of cardiovascular mortality. Depicted are HRs (colored dots) and 95% CIs (colored horizontal lines) for the association of highest compared to lowest reference value of serum IgA (4.76 vs 0.86 g/L), IgG (15.10 vs 6.20 g/L), and IgM (2.64 vs 0.28 g/L) with different types of cardiovascular mortality. HRs are adjusted for age, sex, Rotterdam Study cohort, smoking status, alcohol consumption, physical activity, and highest attained education. 95% CI, 95% confidence interval; HR, hazard ratio; IgA/IgG/IgM, immunoglobulin A/G/M

Participants with highest vs lowest reference value of IgA, and similar values for all other covariates, had higher risks of all cardiovascular (HR: 1.58; 95% CI: 1.23–2.02) and atherosclerotic cardiovascular mortality (HR: 1.63; 95% CI: 1.22–2.19). Similar results were retrieved for the associations of highest vs lowest reference IgG value with the risk of all cardiovascular (HR: 1.65; 95% CI: 1.28–2.11) and atherosclerotic cardiovascular mortality (HR: 1.55; 95% CI: 1.15–2.09) (Fig. 1, Supplementary Table S4). In addition, the highest vs lowest reference value of IgG was associated with an almost twofold increased risk of non-atherosclerotic cardiovascular mortality (Fig. 1, Supplementary Table S4).

In general, effect estimates for the associations between serum Igs and all cardiovascular and atherosclerotic cardiovascular mortality were comparable when stratified by age, sex, or prevalent ACVD status. However, for non-atherosclerotic mortality we reported stronger associations in women than men for higher IgA (HR: 1.24 vs 1.01 per SD) and IgG (HR: 1.25 vs 1.09 per SD). Furthermore, we found stronger associations with non-atherosclerotic mortality in participants ≤ 65 compared to > 65 years for higher IgA (HR: 1.34 vs 1.08 per SD) and IgG (HR: 1.55 vs 1.19 per SD) and in participants without compared to with prevalent ACVD (HR for IgA: 1.18 vs 0.91 per SD; HR for IgG: 1.28 vs 1.09 per SD) (Supplementary Table S5).

Higher IgA was associated with an increased risk of all-cause mortality (HR: 1.09 per SD; 95% CI: 1.05–1.12), as was higher IgG (HR: 1.06 per SD; 95% CI: 1.03–1.10). IgM was not associated with all-cause mortality risk (Supplementary Table S6).

### Association Between Serum Igs and CAC

Higher IgA was associated with severe CAC (odds ratio [OR] per SD: 1.29; 95% CI: 1.03–1.62) (Table 4). Higher IgG was also associated with severe CAC, although not statistically significantly (OR per SD: 1.16; 95% CI: 0.95–1.43). IgM was not associated with CAC score (Table 4).

**Table 4:** Association between immunoglobulins per SD and CAC score categories

		Odds ratio (95% confidence interval)		
	*Total N*	*Model 1*	*Model 2*	*Model 3*
**IgA**				
No calcification (score = 0)	167	Reference	Reference	Reference
Mild calcification (score 0 − 100)	629	1.22 (0.99 − 1.50)	1.29 (1.03 − 1.60)	1.23 (0.99 − 1.53)
Moderate calcification (score 100 − 400)	363	1.23 (0.99 − 1.54)	1.32 (1.05 − 1.67)	1.24 (0.99 − 1.56)
Severe calcification (score > 400)	463	1.27 (1.02 − 1.59)	1.35 (1.08 − 1.70)	1.29 (1.03 − 1.62)
**IgG**				
No calcification (score = 0)	167	Reference	Reference	Reference
Mild calcification (score 0 − 100)	629	1.02 (0.85 − 1.22)	1.06 (0.88 − 1.28)	1.07 (0.88 − 1.29)
Moderate calcification (score 100 − 400)	362	1.07 (0.88 − 1.30)	1.15 (0.94 − 1.41)	1.16 (0.95 − 1.43)
Severe calcification (score > 400)	463	1.08 (0.88 − 1.31)	1.16 (0.94 − 1.42)	1.16 (0.95 − 1.43)
**IgM**				
No calcification (score = 0)	167	Reference	Reference	Reference
Mild calcification (score 0 − 100)	628	1.05 (0.84 − 1.31)	1.06 (0.84 − 1.33)	1.04 (0.81 − 1.35)
Moderate calcification (score 100 − 400)	363	0.96 (0.75 − 1.23)	0.97 (0.75 − 1.25)	0.96 (0.73 − 1.26)
Severe calcification (score > 400)	463	1.02 (0.81 − 1.29)	1.02 (0.80 − 1.31)	1.01 (0.77 − 1.32)

Model 1 is adjusted for age and sex; model 2 is adjusted for model 1, smoking status, alcohol consumption, physical activity, and highest attained education; model 3 is adjusted for model 2, body mass index, diabetes mellitus, hypertension, serum cholesterol, serum C-reactive protein, and serum triglycerides. *CAC* coronary artery calcification, *IgA* immunoglobulin A, *IgG* immunoglobulin G, *IgM* immunoglobulin M, *SD* standard deviation

After exclusion of participants with IgG levels outside the reference range and users of potentially Ig-altering medication, the association of higher IgG with severe CAC was more pronounced (OR per SD: 1.36; 95% CI: 1.00–1.84) (Supplementary Table S7).

## Discussion

In this study, we report that higher serum IgA and IgG levels were associated with an increased risk of all cardiovascular, atherosclerotic, and non-atherosclerotic cardiovascular mortality. Higher serum IgG levels were furthermore associated with an increased risk of ACVD. The reported associations with ACVD and cardiovascular mortality were supported by the association of higher IgA and trend of higher IgG with severe atherosclerosis. Higher serum IgA and IgG were associated with an increased risk of all-cause mortality as well, although less pronounced. Serum IgM was not associated with any of the outcomes. Our reported associations were independent of CRP, which has indirectly been targeted in anti-IL-1 and anti-IL-6 therapy, since these ILs can promote CRP production [30]. This may imply involvement of serum Igs in novel inflammatory processes that could potentially be targeted in patients with a high residual risk of CVD, although more research is needed to investigate this.

Previously, Igs have been detected in the intima of human atherosclerotic plaques [9]. We believe that our results may underline a potential direct effect of Igs on atherosclerosis, ACVD and cardiovascular mortality by binding to atherosclerotic antigens or particles. Vascular calcification, including CAC, is strongly driven by inflammation [31, 32]. Calcifying nanoparticles are thought to play a causal role in vascular calcification, potentially by promoting endothelial dysfunction and downregulation of atheroprotective transcription factors among others [33, 34]. Higher IgG levels directed against calcifying nanoparticles were demonstrated in Turkish individuals with CAC and these IgG levels correlated positively with CAC score [33]. It has been implicated that IgG bound to calcifying nanoparticles can promote further calcification, thus resulting in a vicious loop of inflammation and calcification [35]. Igs may induce or promote atherosclerosis by binding to atherosclerotic antigens as well [8]. Anti-β2-glycoprotein 1 IgA and IgG antibodies have been reported in patients with atherosclerosis and play an important role in atherogenesis, as they facilitate uptake of oxidized low-density lipoprotein by macrophages [36–38]. Antigen-bound IgG can furthermore activate macrophages via Fc receptors and promote foam cell formation, thus inducing plaque formation [39, 40]. Apolipoprotein A1-bound IgG may also inhibit the function of high-density lipoprotein cholesterol [40]. Previously, higher serum IgA levels have been reported in patients with atherosclerosis compared to controls [41]. More recently, IgA antibodies against phosphocholine and *Streptococcus pneumoniae* cell wall polysaccharide were found to be associated with an increased CVD risk, suggesting a potential pathway via gut microbiota and phospholipid synthesis [42]. Igs directed to other pathogens, including *Chlamydia pneumoniae* and *Helicobacter pylori* have been related with atherosclerosis and CVD as well [43, 44]. Possible mechanisms include vascular inflammation due to molecular mimicry or endotoxin-induced increased uptake of lipids by macrophages [44].

The reported associations of serum Igs with ACVD and cardiovascular mortality could partly be explained by underlying atherosclerosis. Higher CAC scores have previously been associated with an increased risk of coronary heart disease, stroke, cardiovascular mortality, cancer related mortality, and all-cause mortality [45, 46]. Interestingly, within the composite outcome of ACVD, we only reported a trend of higher IgG with myocardial infarction and revascularization. HRs for the association with both total and ischemic stroke were around 1, suggesting that the cerebrovascular system is less susceptible for the effects of serum Igs, which may have to do with shielding by the blood brain barrier [47]. However, we also showed an association between higher IgG and non-atherosclerotic cardiovascular mortality, implying that serum Igs are involved in cardiovascular inflammation that bypasses atherosclerosis. A recent Japanese study concluded that Ig free light chain levels were significantly higher in patients with atrial fibrillation, heart failure with sinus rhythm, and hypertrophic cardiomyopathy compared to healthy controls [48]. This suggests that serum Igs may be associated with cardiac disease through pathways other than atherosclerosis as well, future studies should investigate this.

Interestingly, the majority of the associations with cardiovascular mortality were enhanced within the reference range. It is possible that spikes in serum Ig levels are temporary and that chronic low-grade elevated Ig levels on the other hand are clinically relevant with respect to cardiovascular (and potentially other inflammatory) outcomes, as is seen with high-sensitivity CRP levels [49]. Furthermore, participants with reference range Ig levels and without use of potentially Ig-altering medication may have more stable serum Ig levels over time, thus being less likely to suffer from regression towards the mean. We also consistently reported stronger and significant associations of higher IgA and IgG with cardiovascular mortality in participants without baseline ACVD. This may imply that serum Igs could be directly associated with cardiovascular mortality, independent of pre-existing atherosclerosis and ACVD. Furthermore, it may argue against reverse causation (i.e., pre-existing atherosclerotic disease influencing serum Ig levels).

Lastly, we reported associations of higher IgA and IgG levels with all-cause mortality. This is line with the results of a previous population-based study in military men [50]. However, effect estimates for all-cause mortality were smaller than those for cardiovascular mortality, underlining the potentially more prominent contribution of serum Igs to cardiovascular inflammation.

Important strengths or our study include the population-based design with a balanced distribution of men and women, long follow-up with negligible loss to follow-up, and ascertainment of cardiovascular outcomes based on internationally applied clinical criteria and guidelines. We also possessed a wide range of meticulously measured potential confounders. However, our study also knows some limitations. Despite comprehensive adjustment, as with all observational studies, residual confounding cannot be ruled out. We only had serum Ig measurements at baseline, and were therefore unable to associate changes in Ig levels over time with risk of ACVD and mortality. Furthermore, the RS consists of a mainly Caucasian population, possibly limiting extrapolation of our results to other populations. Future studies are warranted to replicate our findings in other populations and to elucidate the underlying pathophysiological pathways and mechanisms. If causality is demonstrated, these pathophysiological processes may be investigated as modifiable treatment target in patients with a residual inflammatory CVD risk. Independent of a causal relationship, applicability of serum Igs as cardiovascular biomarker should be assessed.

## Conclusions

In middle-aged and older individuals from the general population, higher serum IgA and IgG, but not serum IgM, are associated with an increased risk of ACVD, cardiovascular mortality, and severe atherosclerosis. Although we hypothesize a potential causal relationship between serum Igs and cardiovascular morbidity and mortality, future studies are needed to confirm this. Alternatively, utility of serum Igs as biomarker in cardiovascular risk management should be assessed.

## Supplementary Information

Below is the link to the electronic supplementary material.

Supplementary file1 (DOCX 27 KB)

## Data Availability

Data can be obtained upon request. Requests should be directed towards the management team of the Rotterdam Study (datamanagement.ergo@erasmusmc.nl), which has a protocol for approving data requests. Because of restrictions based on privacy regulations and informed consent of the participants, data cannot be made freely available in a public repository.
