# Supplementary material for: The Association of Serum Immunoglobulins with Risk of Cardiovascular Disease and Mortality: the Rotterdam Study
Source: J Clin Immunol. 2023 Feb 1;43(4):769–79. doi: 10.1007/s10875-023-01433-7 (PMC10110646; doi:10.1007/s10875-023-01433-7)
Supplement: Supplementary file 1 — Supplementary file1 (DOCX 27 KB) [file 10875_2023_1433_MOESM1_ESM.docx]

**Supplementary material “The association of serum immunoglobulins with risk of cardiovascular disease and mortality: the Rotterdam Study”**

Samer R. Khan, MD; Virgil A.S.H. Dalm, MD PhD; M. Kamran Ikram, MD PhD; Robin P. Peeters, MD PhD; P. Martin van Hagen, MD PhD; Maryam Kavousi, MD PhD; Layal Chaker, MD PhD

**Corresponding Author**

Layal Chaker, MD PhD

Department of Epidemiology, Erasmus MC

Doctor Molewaterplein 50

3015 GE Rotterdam, the Netherlands

Tel: +31 10 70 43488 / 43391 / 43489

Fax: +31 10 70 44657

Email: l.chaker@erasmusmc.nl

| **Supplementary Table S1. Association between immunoglobulins per SD and risk of ACVD (reference range)^a^** | | | | |
| --- | --- | --- | --- | --- |
| **All** | | | | |
|  |  | **Hazard Ratio (95% Confidence Interval)** | | |
|  | *N events/total* | *Model 1* | *Model 2* | *Model 3* |
| **IgA** | 810/6,481 | 1.04 (0.95-1.13) | 1.06 (0.97-1.16) | 1.06 (0.97-1.16) |
| **IgG** | 803/6,476 | 1.05 (0.96-1.15) | 1.08 (0.99-1.18) | 1.08 (0.99-1.18) |
| **IgM** | 806/6,520 | 1.05 (0.91-1.22) | 1.07 (0.92-1.24) | 1.06 (0.91-1.23) |
| **Myocardial Infarction** | | | | |
|  |  | **Hazard Ratio (95% Confidence Interval)** | | |
|  | *N events/total* | *Model 1* | *Model 2* | *Model 3* |
| **IgA** | 338/6,791 | 0.99 (0.86-1.13) | 1.02 (0.88-1.17) | 1.02 (0.89-1.18) |
| **IgG** | 328/6,792 | 1.04 (0.90-1.19) | 1.07 (0.93-1.23) | 1.07 (0.93-1.23) |
| **IgM** | 332/6,834 | 1.23 (0.99-1.53) | 1.24 (1.00-1.54) | 1.24 (0.99-1.54) |
| **Revascularization** | | | | |
|  |  | **Hazard Ratio (95% Confidence Interval)** | | |
|  | *N events/total* | *Model 1* | *Model 2* | *Model 3* |
| **IgA** | 336/6,770 | 1.03 (0.90-1.19) | 1.07 (0.93-1.23) | 1.05 (0.92-1.21) |
| **IgG** | 334/6,765 | 1.08 (0.94-1.24) | 1.11 (0.97-1.28) | 1.11 (0.96-1.28) |
| **IgM** | 327/6,813 | 0.93 (0.74-1.18) | 0.94 (0.74-1.19) | 0.94 (0.74-1.19) |
| **Stroke** | | | | |
|  |  | **Hazard Ratio (95% Confidence Interval)** | | |
|  | *N events/total* | *Model 1* | *Model 2* | *Model 3* |
| **IgA** | 457/6,865 | 1.04 (0.92-1.17) | 1.06 (0.94-1.19) | 1.05 (0.94-1.19) |
| **IgG** | 456/6,860 | 1.00 (0.89-1.12) | 1.02 (0.91-1.15) | 1.02 (0.91-1.15) |
| **IgM** | 461/6,896 | 1.03 (0.85-1.26) | 1.05 (0.86-1.28) | 1.05 (0.86-1.28) |
| ^a^Comprises reference range values (0.86-4.76 g/L for IgA, 6.20-15.10 g/L for IgG, and 0.28-2.64 g/L for IgM) and exclusion of medication use that can influence serum immunoglobulins (systemic corticosteroids, antiepileptic drugs, angiotensin converting enzyme inhibitors, cytostatics, immunomodulators, and/or immunosuppressants).  Model 1 is adjusted for age, sex, and Rotterdam Study cohort; model 2 is adjusted for model 1, smoking status, alcohol consumption, physical activity, and highest attained education; model 3 is adjusted for model 2, body mass index, diabetes mellitus, hypertension, serum cholesterol, serum C-reactive protein, and serum triglycerides.  ACVD, atherosclerotic cardiovascular disease; IgA, immunoglobulin A; IgG, immunoglobulin G; IgM, immunoglobulin M; SD, standard deviation. | | | | |

| **Supplementary Table S2. Association between immunoglobulins per SD and risk of ischemic stroke** | | | | |
| --- | --- | --- | --- | --- |
|  |  | **Hazard Ratio (95% Confidence Interval)** | | |
|  | *N events/total* | *Model 1* | *Model 2* | *Model 3* |
| **IgA** | 424/8,464 | 1.04 (0.95-1.14) | 1.05 (0.96-1.15) | 1.04 (0.95-1.14) |
| **IgG** | 423/8,454 | 0.98 (0.88-1.08) | 1.01 (0.91-1.11) | 1.01 (0.91-1.12) |
| **IgM** | 424/8,461 | 1.00 (0.92-1.08) | 1.00 (0.92-1.08) | 1.01 (0.93-1.09) |
| Model 1 is adjusted for age, sex, and Rotterdam Study cohort; model 2 is adjusted for model 1, smoking status, alcohol consumption, physical activity, and highest attained education; model 3 is adjusted for model 2, body mass index, diabetes mellitus, hypertension, serum cholesterol, serum C-reactive protein, and serum triglycerides.  IgA, immunoglobulin A; IgG, immunoglobulin G; IgM, immunoglobulin M; SD, standard deviation. | | | | |

| **Supplementary Table S3. Association between immunoglobulins per SD and risk of cardiovascular mortality (assay recommended reference range)^a^** | | | | |
| --- | --- | --- | --- | --- |
| **All cardiovascular mortality** | | | | |
|  |  | **Hazard Ratio (95% Confidence Interval)** | | |
|  | *N events/total* | *Model 1* | *Model 2* | *Model 3* |
| **IgA** | 409/6,866 | 1.16 (1.01-1.33) | 1.18 (1.03-1.35) | 1.18 (1.03-1.35) |
| **IgG** | 409/6,791 | 1.25 (1.11-1.41) | 1.28 (1.14-1.45) | 1.30 (1.15-1.47) |
| **IgM** | 384/6,541 | 1.07 (0.84-1.36) | 1.09 (0.85-1.38) | 1.08 (0.85-1.38) |
| **Atherosclerotic cardiovascular mortality** | | | | |
|  |  | **Hazard Ratio (95% Confidence Interval)** | | |
|  | *N events/total* | *Model 1* | *Model 2* | *Model 3* |
| **IgA** | 288/6,866 | 1.08 (0.91-1.27) | 1.09 (0.93-1.29) | 1.09 (0.93-1.29) |
| **IgG** | 291/6,791 | 1.26 (1.09-1.45) | 1.30 (1.13-1.50) | 1.31 (1.14-1.52) |
| **IgM** | 273/6,541 | 0.96 (0.71-1.29) | 0.98 (0.73-1.32) | 0.98 (0.73-1.31) |
| **Non-atherosclerotic cardiovascular mortality** | | | | |
|  |  | **Hazard Ratio (95% Confidence Interval)** | | |
|  | *N events/total* | *Model 1* | *Model 2* | *Model 3* |
| **IgA** | 121/6,866 | 1.38 (1.08-1.77) | 1.40 (1.09-1.79) | 1.40 (1.09-1.79) |
| **IgG** | 118/6,791 | 1.21 (0.97-1.51) | 1.24 (0.99-1.55) | 1.25 (1.00-1.57) |
| **IgM** | 111/6,541 | 1.35 (0.88-2.06) | 1.36 (0.89-2.07) | 1.35 (0.89-2.06) |
| ^a^Comprises assay recommended reference range values (0.7-4.0 g/L for IgA, 7.0-16.0 g/L for IgG, and 0.4-2.3 g/L for IgM) and exclusion of medication use that can influence serum immunoglobulins (systemic corticosteroids, antiepileptic drugs, angiotensin converting enzyme inhibitors, cytostatics, immunomodulators, and/or immunosuppressants).  Model 1 is adjusted for age, sex, and Rotterdam Study cohort; model 2 is adjusted for model 1, smoking status, alcohol consumption, physical activity, and highest attained education; model 3 is adjusted for model 2, body mass index, diabetes mellitus, hypertension, serum cholesterol, serum C-reactive protein, and serum triglycerides.  IgA, immunoglobulin A; IgG, immunoglobulin G; IgM, immunoglobulin M; SD, standard deviation. | | | | |

| **Supplementary Table S4. Association between highest vs lowest reference value of immunoglobulins and risk of cardiovascular mortality** | | | |
| --- | --- | --- | --- |
| **All cardiovascular mortality** | | | |
|  | **Hazard Ratio (95% Confidence Interval)** | | |
|  | *Model 1* | *Model 2* | *Model 3* |
| **IgA** | 1.58 (1.24-2.01) | 1.62 (1.27-2.05) | 1.58 (1.23-2.02) |
| **IgG** | 1.55 (1.21-1.98) | 1.67 (1.30-2.13) | 1.65 (1.28-2.11) |
| **IgM** | 1.02 (0.90-1.16) | 1.01 (0.89-1.15) | 1.04 (0.91-1.18) |
| **Atherosclerotic cardiovascular mortality** | | | |
|  | **Hazard Ratio (95% Confidence Interval)** | | |
|  | *Model 1* | *Model 2* | *Model 3* |
| **IgA** | 1.61 (1.21-2.15) | 1.64 (1.23-2.18) | 1.63 (1.22-2.19) |
| **IgG** | 1.42 (1.05-1.92) | 1.55 (1.15-2.10) | 1.55 (1.15-2.09) |
| **IgM** | 1.03 (0.89-1.19) | 1.01 (0.86-1.17) | 1.04 (0.89-1.21) |
| **Non-atherosclerotic cardiovascular mortality** | | | |
|  | **Hazard Ratio (95% Confidence Interval)** | | |
|  | *Model 1* | *Model 2* | *Model 3* |
| **IgA** | 1.50 (0.96-2.34) | 1.52 (0.97-2.38) | 1.45 (0.91-2.29) |
| **IgG** | 1.89 (1.22-2.94) | 1.96 (1.27-3.03) | 1.91 (1.22-2.97) |
| **IgM** | 1.01 (0.79-1.27) | 1.01 (0.79-1.29) | 1.02 (0.80-1.31) |
| Model 1 is adjusted for age, sex, and Rotterdam Study cohort; model 2 is adjusted for model 1, smoking status, alcohol consumption, physical activity, and highest attained education; model 3 is adjusted for model 2, body mass index, diabetes mellitus, hypertension, serum cholesterol, serum C-reactive protein, and serum triglycerides.  Highest vs lowest reference values were 4.76 vs 0.86 g/L for IgA, 15.10 vs 6.20 g/L for IgG, and 2.64 vs 0.28 g/L for IgM. In the comparisons, median values were included for the continuous covariates and the reference category was included for the categorical covariates.  IgA, immunoglobulin A; IgG, immunoglobulin G; IgM, immunoglobulin M. | | | |

| **Supplementary Table S5. Stratifications for the association between immunoglobulins per SD and risk of cardiovascular mortality** | | | | | | | |
| --- | --- | --- | --- | --- | --- | --- | --- |
| **All cardiovascular mortality** | | | | | | | |
|  |  | **IgA** | | **IgG** | | **IgM** | |
| **Sex** | | *N events/total* | *HR (95% CI)* | *N events/total* | *HR (95% CI)* | *N events/total* | *HR (95% CI)* |
|  | *Men* | 327/3,773 | 1.05 (0.95-1.16) | 327/3,770 | 1.16 (1.04-1.29) | 327/3,771 | 1.03 (0.98-1.09) |
|  | *Women* | 328/4,993 | 1.21 (1.10-1.32) | 328/4,986 | 1.12 (1.03-1.22) | 328/4,991 | 0.95 (0.85-1.07) |
| **Age** | |  |  |  |  |  |  |
|  | *≤65 years* | 81/5,283 | 1.26 (1.05-1.52) | 81/5,276 | 1.28 (1.02-1.61) | 81/5,281 | 1.15 (0.89-1.50) |
|  | *>65 years* | 574/3,483 | 1.16 (1.08-1.25) | 574/3,480 | 1.19 (1.11-1.28) | 574/3,481 | 1.01 (0.95-1.07) |
| **Prevalent ACVD** | |  |  |  |  |  |  |
|  | *No* | 449/7,852 | 1.16 (1.07-1.26) | 449/7,842 | 1.19 (1.09-1.30) | 449/7,849 | 1.01 (0.95-1.08) |
|  | *Yes* | 197/822 | 1.02 (0.89-1.16) | 197/822 | 1.08 (0.97-1.20) | 197/821 | 1.07 (0.94-1.21) |
| **Atherosclerotic cardiovascular mortality** | | | | | | | |
|  |  | **IgA** | | **IgG** | | **IgM** | |
| **Sex** | | *N events/total* | *HR (95% CI)* | *N events/total* | *HR (95% CI)* | *N events/total* | *HR (95% CI)* |
|  | *Men* | 208/3,773 | 1.08 (0.95-1.22) | 208/3,770 | 1.20 (1.05-1.36) | 208/3,771 | 1.04 (0.98-1.11) |
|  | *Women* | 247/4,993 | 1.20 (1.08-1.33) | 247/4,986 | 1.07 (0.97-1.19) | 247/4,991 | 0.93 (0.82-1.06) |
| **Age** | |  |  |  |  |  |  |
|  | *≤65 years* | 44/5,283 | 1.14 (0.84-1.56) | 44/5,276 | 1.05 (0.75-1.47) | 44/5,281 | 1.17 (0.82-1.70) |
|  | *>65 years* | 411/3,483 | 1.20 (1.10-1.30) | 411/3,480 | 1.19 (1.09-1.29) | 411/3,481 | 1.02 (0.95-1.09) |
| **Prevalent ACVD** | |  |  |  |  |  |  |
|  | *No* | 325/7,852 | 1.15 (1.05-1.27) | 325/7,842 | 1.15 (1.04-1.28) | 325/7,849 | 1.01 (0.93-1.09) |
|  | *Yes* | 124/822 | 1.07 (0.91-1.26) | 124/822 | 1.08 (0.94-1.22) | 124/821 | 1.11 (0.97-1.28) |
| **Non-atherosclerotic cardiovascular mortality** | | | | | | | |
|  |  | **IgA** | | **IgG** | | **IgM** | |
| **Sex** | | *N events/total* | *HR (95% CI)* | *N events/total* | *HR (95% CI)* | *N events/total* | *HR (95% CI)* |
|  | *Men* | 119/3,773 | 1.01 (0.84-1.20) | 119/3,770 | 1.09 (0.91-1.31) | 119/3,771 | 1.00 (0.89-1.14) |
|  | *Women* | 81/4,993 | 1.24 (1.03-1.48) | 81/4,986 | 1.25 (1.08-1.45) | 81/4,991 | 1.02 (0.82-1.28) |
| **Age** | |  |  |  |  |  |  |
|  | *≤65 years* | 37/5,283 | 1.34 (1.07-1.68) | 37/5,276 | 1.55 (1.16-2.07) | 37/5,281 | 1.15 (0.79-1.69) |
|  | *>65 years* | 163/3,483 | 1.08 (0.93-1.24) | 163/3,480 | 1.19 (1.04-1.36) | 163/3,481 | 0.99 (0.88-1.12) |
| **Prevalent ACVD** | |  |  |  |  |  |  |
|  | *No* | 124/7,852 | 1.18 (1.01-1.37) | 124/7,842 | 1.28 (1.10-1.50) | 124/7,849 | 1.02 (0.91-1.15) |
|  | *Yes* | 73/822 | 0.91 (0.72-1.16) | 73/822 | 1.09 (0.90-1.32) | 73/821 | 0.99 (0.77-1.27) |
| HRs are adjusted for age and/or sex (depending on the stratification), Rotterdam Study cohort, smoking status, alcohol consumption, physical activity, highest attained education, body mass index, diabetes mellitus, hypertension, serum cholesterol, serum C-reactive protein, and serum triglycerides.  95% CI, 95% confidence interval; ACVD, atherosclerotic cardiovascular disease (comprises myocardial infarction, revascularization, or stroke); HR, hazard ratio; IgA, immunoglobulin A; IgG, immunoglobulin G; IgM, immunoglobulin M; SD, standard deviation. | | | | | | | |

| **Supplementary Table S6. Association between immunoglobulins per SD and risk of all-cause mortality** | | | | |
| --- | --- | --- | --- | --- |
|  |  | **Hazard Ratio (95% Confidence Interval)** | | |
|  | *N events/total* | *Model 1* | *Model 2* | *Model 3* |
| **IgA** | 2,991/8,766 | 1.09 (1.06-1.13) | 1.10 (1.07-1.14) | 1.09 (1.05-1.12) |
| **IgG** | 2,989/8,756 | 1.05 (1.01-1.08) | 1.07 (1.03-1.10) | 1.06 (1.03-1.10) |
| **IgM** | 2,990/8,762 | 1.01 (0.98-1.03) | 1.00 (0.97-1.03) | 1.00 (0.97-1.03) |
| Model 1 is adjusted for age, sex, and Rotterdam Study cohort; model 2 is adjusted for model 1, smoking status, alcohol consumption, physical activity, and highest attained education; model 3 is adjusted for model 2, body mass index, diabetes mellitus, hypertension, serum cholesterol, serum C-reactive protein, and serum triglycerides.  IgA, immunoglobulin A; IgG, immunoglobulin G; IgM, immunoglobulin M; SD, standard deviation. | | | | |

| **Supplementary Table S7. Association between immunoglobulins per SD and CAC score categories (reference range)^a^** | | | | |
| --- | --- | --- | --- | --- |
|  |  | **Odds Ratio (95% Confidence Interval)** | | |
|  | *Total N* | *Model 1* | *Model 2* | *Model 3* |
| **IgA** |  |  |  |  |
| No calcification (score = 0) | 148 | REFERENCE | REFERENCE | REFERENCE |
| Mild calcification (score 0-100) | 534 | 1.10 (0.85-1.41) | 1.14 (0.88-1.48) | 1.02 (0.78-1.34) |
| Moderate calcification (score 100-400) | 298 | 1.33 (1.01-1.74) | 1.41 (1.06-1.86) | 1.25 (0.94-1.68) |
| Severe calcification (score >400) | 337 | 1.20 (0.91-1.59) | 1.27 (0.96-1.70) | 1.15 (0.85-1.54) |
| **IgG** |  |  |  |  |
| No calcification (score = 0) | 147 | REFERENCE | REFERENCE | REFERENCE |
| Mild calcification (score 0-100) | 530 | 1.15 (0.89-1.47) | 1.18 (0.91-1.53) | 1.15 (0.87-1.50) |
| Moderate calcification (score 100-400) | 297 | 1.29 (0.98-1.70) | 1.40 (1.05-1.86) | 1.37 (1.02-1.84) |
| Severe calcification (score >400) | 332 | 1.27 (0.96-1.68) | 1.39 (1.04-1.87) | 1.36 (1.00-1.84) |
| **IgM** |  |  |  |  |
| No calcification (score = 0) | 145 | REFERENCE | REFERENCE | REFERENCE |
| Mild calcification (score 0-100) | 531 | 0.98 (0.67-1.42) | 1.03 (0.70-1.52) | 1.08 (0.72-1.62) |
| Moderate calcification (score 100-400) | 297 | 0.88 (0.57-1.35) | 0.95 (0.61-1.48) | 0.99 (0.63-1.55) |
| Severe calcification (score >400) | 337 | 1.11 (0.72-1.71) | 1.18 (0.76-1.85) | 1.19 (0.75-1.89) |
| ^a^Comprises reference range values (0.86-4.76 g/L for IgA, 6.20-15.10 g/L for IgG, and 0.28-2.64 g/L for IgM) and exclusion of medication use that can influence serum immunoglobulins (systemic corticosteroids, antiepileptic drugs, angiotensin converting enzyme inhibitors, cytostatics, immunomodulators, and/or immunosuppressants).  Model 1 is adjusted for age and sex; model 2 is adjusted for model 1, smoking status, alcohol consumption, physical activity, and highest attained education; model 3 is adjusted for model 2, body mass index, diabetes mellitus, hypertension, serum cholesterol, serum C-reactive protein, and serum triglycerides.  CAC, coronary artery calcification; IgA, immunoglobulin A; IgG, immunoglobulin G; IgM, immunoglobulin M; SD, standard deviation. | | | | |
